# Time for a change of practice: the real-world value of testing for neuronal autoantibodies in acute first-episode psychosis

**DOI:** 10.1192/bjo.2018.27

**Published:** 2018-07-16

**Authors:** Thomas A. Pollak, Belinda R. Lennox

**Affiliations:** Wellcome Trust Clinical Research Training Fellow, Department of Psychosis Studies, Institute of Psychiatry, Psychology and Neuroscience, Kings College London, UK; Clinical Senior Lecturer, Department of Psychiatry, University of Oxford, UK

## Abstract

It is time that all patients with acute-onset psychosis are screened for autoimmune encephalitis, that lumbar puncture becomes a routine psychiatric investigation and that immunotherapy is available in indicated cases. We call for a culture change in the management of psychosis by psychiatry.

**Declaration of interest:**

None.

In 2007, the description of N-Methyl-D-Aspartate Receptor (NMDAR) antibody encephalitis^1^ and its subsequent recognition as a leading cause of autoimmune encephalitis has been a game changer for clinical neurology. This progressive multistage encephalopathy, caused by immunoglobulin G (IgG) autoantibodies to the NR1 subunit of the NMDAR, usually presents with psychosis before evolving into a complex, polymorphic ‘neurological’ presentation involving seizures, amnestic disturbance, movement disorder and autonomic dysfunction. Initial series reported that nearly 80% of patients were initially seen by mental health services,^2^ and often it is only the development of neurological symptoms that alerts clinicians to an organic basis for the patient's symptoms. The importance of early detection of the disorder and aggressive treatment with immunotherapies are now accepted by neurology colleagues worldwide who are vigilant to the possibility of this disorder.^3^

Subsequent reports of ‘isolated psychiatric’ cases of NMDAR antibody encephalitis that were nonetheless responsive to the usual immunotherapies (intravenous corticosteroids, intravenous immunoglobulins, plasma exchange and second-line therapies such as rituximab and mycophenolate mofetil)^4^^,^^5^ caught the psychiatric imagination. A flurry of papers followed, all aiming to characterise the prevalence of NMDAR and other neuronal autoantibodies (usually implicated in other forms of autoimmune encephalitis) in populations of patients with psychotic disorders.^6^ The implication, sometimes incompletely articulated, is that the presence of these antibodies might demarcate either autoimmune encephalitis caught early, or a *forme fruste* of autoimmune encephalitis, ‘misdiagnosed’ as a primary psychiatric disorder such as schizophreniform disorder (we use quotation marks to acknowledge that psychiatric diagnostic systems reflect symptoms rather than aetiology, and existing categories are likely to be aetiologically heterogenous). Taken together, these two categories might represent a subset of patients in psychiatric wards and clinics that require a very different approach to assessment and treatment.

However, to date these studies and findings have not transformed psychiatric clinical practice as recognition of autoimmune encephalitis has transformed that of our neurological colleagues. This is partly because of the differences in our infrastructures. It is straightforward for a disorder that requires investigations such as magnetic resonance imagining (MRI), a lumbar puncture and an electroencephalogram (EEG) to become part of the diagnostic landscape for neurologists, who have these tools readily available. Too often patients in psychiatric units with a positive serum neuronal autoantibody test result will not receive these essential supporting investigations at all, and psychiatric treatments continue as usual: the magnitude of the problem remains unknown.

To add to the uncertainty, there are now reports of seropositivity in a wide range of conditions, as well as in healthy controls, so a further possibility is raised that the antibodies are epiphenomenal and not actually relevant to the clinical presentation at all. Further complexity still revolves around the varying definition of a ‘positive’ antibody test, dependent on the bodily fluid tested, the type of assay used and the use of confirmatory immunological tests. Most clinicians can agree, however, that a positive serum neuronal autoantibody test must be supplemented by EEG, MRI and, essentially, cerebrospinal fluid (CSF) analysis before the relevance of that result is clear.

However, the most pressing questions for psychiatrists are clinical ones: should we screen patients with psychotic disorders for the antibodies, and do patients with psychotic disorders and neuronal autoantibodies respond to immunotherapy?

In this issue, Scott *et al* contribute important clinical data to help address these questions.^7^ They undertook prospective testing for serum neuronal autoantibodies in 113 patients aged between 12 and 50 years, presenting with a first episode of psychosis in three mental health hospitals in Queensland, Australia. Importantly, samples were collected even in the most unwell patients, with retrospective consent sought after capacity was regained. They managed to include a high proportion (73%) of eligible patients. This sets the study apart from most other antibody prevalence studies, which have utilised either research cohorts (who are capacitous, prescreened for organic red flags and often in at least partial remission) or community cohorts.

They found that six participants (5.3%) tested positive for serum neuronal autoantibodies, four with NMDAR antibodies, one with low-titre voltage-gated potassium channel (VGKC) antibodies and serum from a final participant demonstrated staining of cerebellar tissue but without a specific antigen identified after testing for some of the more likely possibilities. Four of these patients were the most acutely unwell, and non-capacitous at the time of testing, with an abrupt onset to their illness (i.e. a duration of untreated psychosis of a week or less). Two patients with NMDAR antibodies progressed to develop a classical NMDAR antibody encephalopathy, with seizures along with detection of a teratoma: they are therefore examples of NMDAR antibody encephalitis caught early. The other three patients (two with NMDAR antibodies and one with low-titre VGKC antibodies) had isolated psychiatric presentations, potentially representing the kind of *forme fruste* described above. All had normal MRI results. Crucially, all four patients who were seropositive for NMDAR antibody had a lumbar puncture and CSF analysis performed: CSF NMDAR antibodies were detected in three patients, but all had evidence of a CSF inflammatory process. The patients who were seropositive for NMDAR and VGKC antibodies received immunotherapy, consisting of intravenous methylprednisolone, intravenous immunoglobulins and in three patients, a second-line or maintenance treatment (rituximab or azathioprine). All four patients with NMDAR antibodies showed a sustained improvement following immunotherapy, including remission of psychosis.

Returning to the questions above, we believe the answer to the first question is yes, all in-patients with an acute-onset psychosis (duration of untreated psychosis of 3 months or less) should be screened for NMDAR antibodies. This would be in keeping with screening guidelines for NMDAR antibody encephalitis.^7^ The fact that all four patients who were seropositive for NMDAR antibodies had convincing ancillary evidence of an active central nervous system autoimmune process and likely positive immunotherapy response suggests that screening for NMDAR antibodies in acute first episode psychosis is of clear value and may, after appropriate further investigation, result in treatment that is potentially life-saving or at the very least disease-modifying. Indeed, given a 3.8% detection rate of NMDAR antibodies that have clear pathogenic relevance regardless of whether the patient in question met strict criteria for definite NMDAR antibody encephalitis^8^ (as only three of four patients did), it would appear to be neglectful not to do so.

Regarding the second question, that of immunotherapy response, it is crucial to note that even the patients with the wholly psychiatric presentation responded to immunotherapy. Yet the two patients with NMDAR antibodies who had an isolated psychiatric presentation would not have attracted clinical suspicion were it not for antibody screening, having received initial diagnoses of schizophreniform disorder and bipolar affective disorder. The implication, essential for psychiatrists, is that we cannot rely on clinical history or symptomatic presentation (so-called ‘red flags’) to rule out an immunotherapy responsive disorder. Outside of a trial setting, it is not possible to say whether the patients who improved with immunotherapy may have got better anyway, as the majority of patients with first episode psychosis will improve with antipsychotic medication alone. But the presence of inflammatory CSF changes would, for most clinicians, exclude psychiatric treatment-as-usual as a defensible option.

(The patient with VGKC antibodies appeared to respond to immunotherapy but had a relapsing course of illness subsequently. We now know, although the authors did not at the time of the study, that VGKC antibodies, in the absence of antibodies to the associated proteins LGI1 and CASPR2, are unlikely to be pathogenic:^9^ this may explain the patient's clinical course.)

It is worth emphasising that a positive NMDAR antibody serum test does not equate to a diagnosis of NMDAR antibody encephalitis,^8^ nor does it imply on its own that a given patient will respond to immunotherapy: it is important therefore that all patients who test positive should have a lumbar puncture with CSF analysed not just for NMDAR antibodies but for other markers of inflammation, including white blood cells, protein and oligoclonal bands, as well as MRI and EEG, to enable an informed decision about treatment with immunotherapy.

Scott *et al*^7^ point out in their discussion that the rate of detection of central nervous system autoimmunity in their study may have been underestimated as CSF analysis was only undertaken on patients who were seropositive for neuronal autoantibodies. Indeed, they recommend CSF testing in seronegative patients with acute psychosis who have signs and symptoms suggestive of autoimmune encephalitis. We agree that this is a sensible suggestion, but one that does not go far enough. Despite the fact that CSF testing does not feature in current guidelines for the management of psychosis (e.g. www.nice.org.uk; www.dgppn.de), an evidence base is emerging demonstrating that routine CSF analysis in patients with psychosis does reveal markers of inflammatory or infective aetiologies, in addition to the detection of autoimmune encephalitis. In some of these cases the CSF findings directly lead to re-diagnosis and/or an alternative treatment approach.^10^^–^^14^ These findings indicate that lumbar punctures may, in this context, show more clinical utility than other investigations such as MRI.^15^^–^^17^

For this reason, we would go one step further than Scott *et al*^7^ and recommend that lumbar puncture is offered to all patients with new-onset acute psychosis as part of the broader evaluation of their mental and physical health. In centres where this is routinely undertaken, there is a high level of acceptability and tolerability of the procedure.^11^^,^^13^ We suggest that the time for cultural change in psychiatric practice may be upon us.
